# Exercise intervention improves mitochondrial quality in non-alcoholic fatty liver disease zebrafish

**DOI:** 10.3389/fendo.2023.1162485

**Published:** 2023-05-22

**Authors:** Yun-Yi Zou, Xiang-bin Tang, Zhang-Lin Chen, Bin Liu, Lan Zheng, Ming-Yang Song, Qin Xiao, Zuo-Qiong Zhou, Xi-Yang Peng, Chang-Fa Tang

**Affiliations:** State Key Laboratory of Developmental Biology of Freshwater Fish, Key Laboratory of Physical Fitness and Exercise Rehabilitation of Hunan Province, College of Physical Education, Hunan Normal University, Changsha, China

**Keywords:** exercise, non-alcoholic fatty liver disease, mitochondria quality control, zebrafish, mitochondrial dysfunction

## Abstract

**Introduction:**

Recent reports indicate that mitochondrial quality decreases during non-alcoholic fatty liver disease (NAFLD) progression, and targeting the mitochondria may be a possible treatment for NAFLD. Exercise can effectively slow NAFLD progression or treat NAFLD. However, the effect of exercise on mitochondrial quality in NAFLD has not yet been established.

**Methods:**

In the present study, we fed zebrafish a high-fat diet to model NAFLD, and subjected the zebrafish to swimming exercise.

**Results:**

After 12 weeks, swimming exercise significantly reduced high-fat diet-induced liver injury, and reduced inflammation and fibrosis markers. Swimming exercise improved mitochondrial morphology and dynamics, inducing upregulation of optic atrophy 1(OPA1), dynamin related protein 1 (DRP1), and mitofusin 2 (MFN2) protein expression. Swimming exercise also activated mitochondrial biogenesis via the sirtuin 1 (SIRT1)/ AMP-activated protein kinase (AMPK)/ PPARgamma coactivator 1 alpha (PGC1α) pathway, and improved the mRNA expression of genes related to mitochondrial fatty acid oxidation and oxidative phosphorylation. Furthermore, we find that mitophagy was suppressed in NAFLD zebrafish liver with the decreased numbers of mitophagosomes, the inhibition of PTEN-induced kinase 1 (PINK1) – parkin RBR E3 ubiquitin protein ligase (PARKIN) pathway and upregulation of sequestosome 1 (P62) expression. Notably, swimming exercise partially recovered number of mitophagosomes, which was associated with upregulated PARKIN expression and decreased p62 expression.

**Discussion:**

These results demonstrate that swimming exercise could alleviate the effects of NAFLD on the mitochondria, suggesting that exercise may be beneficial for treating NAFLD.

## 1 Introduction

Poor dietary habits are an emerging health problem, and are linked to the development of metabolic syndromes, including non-alcoholic fatty liver disease (NAFLD) (1). NAFLD is characterized by excessive fat deposition in hepatic cells. Surplus hepatocellular lipids contribute to lipotoxicity, oxidative stress, inflammation, and fibrosis in the liver, and are closely linked to the development of hepatocellular carcinoma (HCC) (2). NAFLD is often accompanied by various extrahepatic complications, such as type 2 diabetes and cardiovascular events (3). The prevalence of NAFLD is increasing, and it is now one of the major financial and medical burdens globally, with adverse consequences for society and for individual quality of life. Finding preventive measures and effective therapeutic strategies for slowing down or treating NAFLD could alleviate the global burden of this disease.

The liver is the major organ where regulation of carbon metabolism (glucose, lipids, and protein) occurs, and mitochondria are vital organelles in hepatic metabolic pathways and signaling networks (4). Lipid catabolism mainly depends on mitochondrial fatty acid β-oxidation. In NAFLD, mitochondrial biogenesis, which is regulated by PGC1α, is strongly suppressed, resulting in reduced mitochondrial oxidative phosphorylation (OXPHOS), mitochondrial respiration, and β-oxidation (5, 6), thus enhancing fat accumulation and driving disease progression. Mitochondria are dynamic organelles, and regular fission and fusion are necessary to maintain their size and morphology. However, under pathological NAFLD conditions, fission-related factor DRP1 and fusion-related factors MFN2 and OPA1 are dysregulated (7–9). Significantly smaller mitochondria with increased mass have been found in fatty liver tissue (5). In addition to morphological changes, NAFLD may affect the process of mitophagy, which is the classical process for removing damaged mitochondria by autophagy. The expression of genes related to autophagy is reduced in NAFLD patients, and the activation of mitophagy could therefore protect against the progression of NAFLD (8, 10). In addition, mitochondria are the major cellular source of reactive oxygen species (ROS) (11). High levels of ROS are associated with apoptosis (12), and have an important role in the development of NAFLD (13). Since a decrease in mitochondrial quality is associated with NAFLD, targeting mitochondria could ameliorate the progression of NAFLD.

Regular exercise is an effective measure for treating or delaying the progression of NAFLD (14, 15) by reducing intrahepatic fat deposition, increasing β-oxidation, suppressing ROS production, and attenuating hepatocyte apoptosis (16). Exercise could also regulate anti-inflammatory factors to improve the inflammatory response in NAFLD (14). Moreover, exercise may have an ameliorative effect on liver fibrosis in NAFLD patients (17). However, the effect of exercise on the mitochondria in NAFLD remains unknown. In the present study, we used a high-fat diet to model NAFLD in zebrafish, and exerted swimming exercise on NAFLD model zebrafish to explore the role of exercise in fatty liver disease and investigate associated improvements in mitochondrial quality.

## 2 Materials and methods

### 2.1 Animal models

Zebrafish were fed a high-fat diet to induce NAFLD as previously described (16). Six month-old AB strain zebrafish were raised under 14 h of light at 28°C under standard husbandry conditions. Zebrafish were randomly divided into three groups (*n* = 21 zebrafish/group): normal diet (N), high-fat diet (H), and high-fat diet plus exercise (HE). The N group zebrafish were fed a low-fat diet containing 6% fat (TP1FM21051, Trophic Animal Feed High-Tech Co., Ltd., Nantong, Jiangsu Province, China) for 12 weeks. The H group zebrafish received a high-fat diet containing 24% fat (TP1FM21050, Trophic Animal Feed High-Tech Co., Ltd., Nantong, Jiangsu Province, China) for 12 weeks. The HE group zebrafish received the same high-fat diet as the H group, but were also subjected to swimming exercise. After 12 weeks, zebrafish were anesthetized with tricaine for the collection of tissue samples. This research was conducted in accordance with the Chinese guidelines for animal welfare and experimental protocols. Approval was obtained from the Animal Experiment Administration Committee of Hunan Normal University (Changsha, China) (approval number: 2018/046).

### 2.2 Exercise protocol

Swimming exercise was performed as previously described (16). Briefly, for the first month, HE group zebrafish were placed in a tank with a water current causing them to swim at a 6× body length (BL)/s swimming speed (16 cm/s, approximately 40% Ucrit). For the next 2 months, the HE group zebrafish were placed in a tank with a water current causing them to swim at 8× BL/s (22 cm/s, ~55% Ucrit). During periods of exercise, zebrafish in the HE group were moved to a swimming tunnel, acclimated for 30 min, and exercised for 4 h per day. Zebrafish were exercised for 5 days per week for the duration of the experiment.

### 2.3 Histological analysis

Three biological replicates were used for H&E staining, MASSON staining and DHE staining. Hematoxylin and eosin (H&E) staining was conducted on formalin-fixed paraffin embedded (FFPE) liver tissue sections for the evaluation of pathological changes. The NAFLD activity score (NAS) was calculated according to the guidelines provided by the Pathology Committee of the NASH Clinical Research Network (18). MASSON staining was conducted on FFPE liver tissue sections for the evaluation of fibrosis progression. DHE staining was conducted on frozen liver tissue sections for the evaluation of ROS accumulation.

### 2.4 Transmission electron microscopy

To characterize the mitochondrial ultrastructure, liver tissue was analyzed by transmission electron microscopy (TEM), as described previously (16), two biological replicates were used for TEM. Quantification of mitochondrial parameters (number, diameter, length, and size) in hepatocytes was performed using ImageJ (19). The morphology of mitophagosomes was characterized according to previous studies (20, 21).

### 2.5 Quantitative real time-PCR

Six biological replicates were used for real-time qPCR. Total RNA was extracted from liver tissue samples using TRIzol reagent (Thermo Fisher Scientific, Waltham, MA, USA) according to the manufacturer’s instructions. RNA was reverse-transcribed into cDNA using the PrimeScript™ RT reagent Kit with gDNA Eraser (Takara, Tokyo, Japan). qPCR was conducted using SYBR Green Master Mix (Thermo Fisher Scientific). The relative mRNA expression of target genes was determined using a Bio-Rad real-time PCR system (CFX96; Bio-Rad Laboratories, Hercules, CA, USA). The 2^−ΔΔCT^ method was used to calculate the relative mRNA expression, with *gapdh* used as the reference gene. Primers were synthesized by Sangon Biotech. Primer sequences are shown in **Supplementary Table 1**.

### 2.6 Western blotting

Total protein was extracted in lysis buffer supplemented with protease and phosphatase inhibitors (Solarbio, Wuhan, China). Western blotting was carried out according to our previous publication (22), six zebrafish liver samples in each group were performed for *Western bloting.* The antibodies used were as follows: rabbit anti-GAPDH antibody (1:2000; servicebio), rabbit anti-COL1A1 antibody (1:1000; Wanleibio), rabbit anti-ACTA2 antibody (1:1000; Proteintech), rabbit anti-IL-1β antibody (1:2000; Wanleibio), mouse anti-IL10 antibody (1:1500; Proteintech), rabbit anti-DRP1 antibody (1:1500; Proteintech), rabbit anti-OPA1 antibody (1:1500; Proteintech), rabbit anti-MFN2 antibody (1:1500; Proteintech), rabbit anti-P-AMPK antibody (1:2000; Cell Signaling Technology), rabbit anti-AMPK antibody (1:1500; Proteintech), rabbit anti-PGC1α antibody (1:1000; Bioss), rabbit anti-NRF1 antibody (1:1000; Proteintech), rabbit anti-NRF2 antibody (1:2000; Proteintech), rabbit anti-TFAM antibody (1:2000; Proteintech), rabbit anti-PINK1antibody (1:2000; Proteintech), rabbit anti-PARKIN antibody (1:2000; Bioss) and rabbit anti-P62 antibody (1:2000; Proteintech). Protein expression level was normalized to that of GAPDH.

### 2.7 Statistical analysis

All statistical analyses were performed using GraphPad Prism 9.0 (San Diego, CA, USA). Differences between groups were assessed using one-way analysis of variance (ANOVA) and a Tukey *post hoc* test. Differences were considered significant at *p* ≤ 0.05. Values are expressed as the mean ± standard error.

## 3 Results

### 3.1 Swimming exercise prevents the progression of diet-induced NAFLD in zebrafish

**Figure 1:**
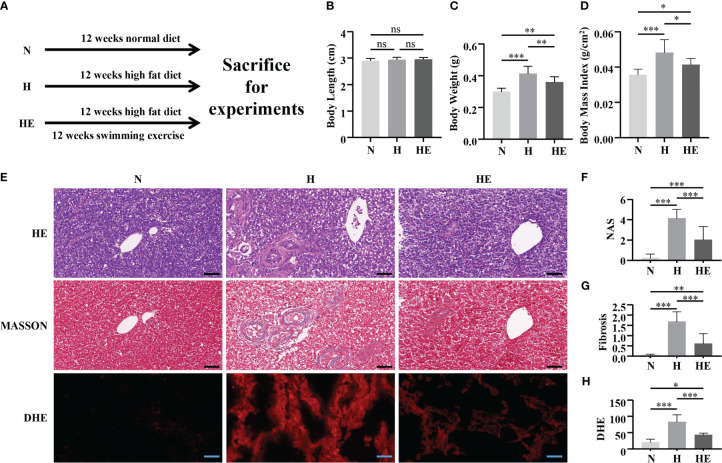
Protective effect of swimming exercise against diet-induced NAFLD in zebrafish. **(A)** Schematic of the swimming exercise strategy to study diet-induced NAFLD in zebrafish. **(B)** Body length, **(C)** body weight, and **(D)** body mass index of zebrafish during the experimental period. **(E)** HE staining, MASSON staining, and DHE visualization of zebrafish liver tissue in response to a high-fat diet and a high-fat diet combined with exercise (n=3). **(F)** NAS score, **(G)** fibrosis score, and **(H)** DHE integrated density in zebrafish livers. *, *p* < 0.05, **, *p* < 0.01, ***, *p* < 0.001. Data represent the mean, and error bars represent the SEM. Scale bar, 20 μm. NAFLD, non-alcoholic fatty liver disease; N, normal diet; H, high fat diet; HE, high-fat diet plus exercise. ns, not significant.

The therapeutic effect of swimming exercise on the pathological progression of NAFLD in zebrafish fed a high-fat diet was investigated (**Figure 1A**). Although zebrafish body length did not change between the three groups (**Figure 1B**), a high-fat diet significantly increased zebrafish body weight and body mass index, while exercise reduced this increase (**Figures 1C, D**). Notably, swimming exercise dramatically attenuated the pathological features of NAFLD (**Figure 1E**), including the NAS score (**Figure 1F**), fibrosis progression (**Figure 1G**), and ROS accumulation (**Figure 1H**). These data suggest that swimming exercise has a protective effect against the progression of diet-induced NAFLD.

### 3.2 Swimming exercise inhibits the expression of inflammation and fibrosis markers in diet-induced NAFLD zebrafish livers

**Figure 2:**
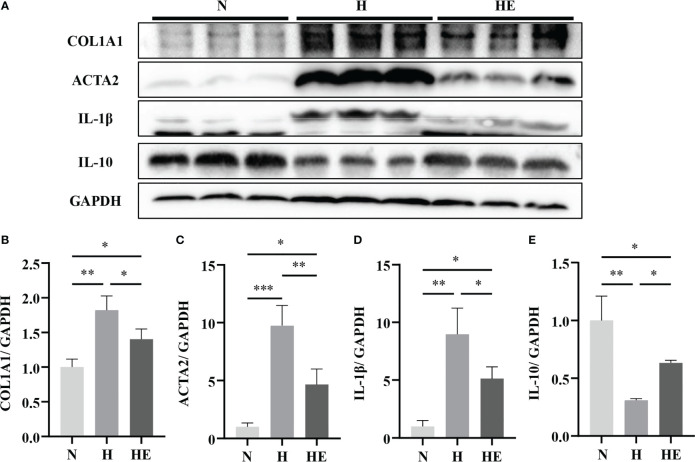
Swimming exercise protects against inflammation and fibrosis in diet-induced NAFLD zebrafish livers. **(A)** Western-blot of CLO1A1, ACTA2, IL-1β, and IL-10. Protein expression levels of **(B)** COL1A1, **(C)** ACTA2, **(D)** IL-1β, and **(E)** IL-10. (n=6) *, *p* < 0.05, **, *p* < 0.01, ***, *p* < 0.001. Data represent the mean, and error bars represent SEM. Scale bar, 20 μm. NAFLD, non-alcoholic fatty liver disease; N, normal diet; H, high fat diet; HE, high-fat diet plus exercise.

To confirm that swimming exercise has a beneficial effect on NAFLD progression, we measured the expression of fibrosis (COL1A1 and ACTA2) and inflammation markers (IL-β) in high-fat diet-induced NAFLD zebrafish livers. As show in **Figure 2**, hepatic fibrosis markers (COL1A1 and ACTA2) and inflammation markers (IL-β) were markedly decreased and the anti-inflammatory cytokine (IL-10) was increased in the HE group compared to in the H group. Swimming exercise can therefore attenuate inflammation and slow the progress of fibrosis in diet-induced NAFLD zebrafish livers.

### 3.3 Swimming exercise improves mitochondrial morphology and dynamics in diet-induced NAFLD zebrafish

**Figure 3:**
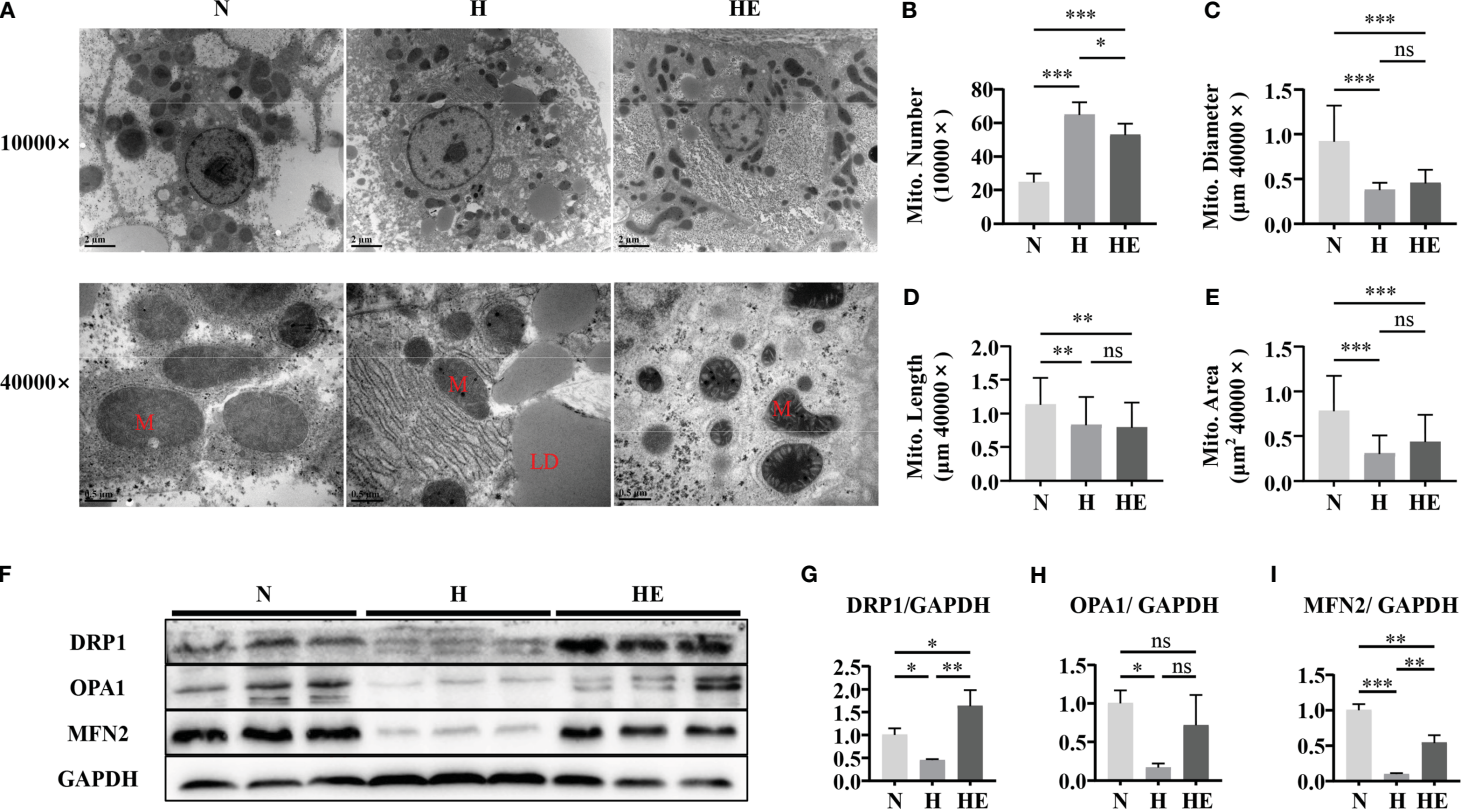
Swimming exercise maintains mitochondrial dynamic balance in diet-induced NAFLD zebrafish livers. **(A)** Representative TEM of zebrafish liver showing visible LDs in fish fed a high-fat diet. TEM micrographs were used to determine **(B)** mitochondrial numbers at 10000× magnification, **(C)** mitochondrial diameter at 40000× magnification, **(D)** mitochondrial length at 40000× magnification, and **(E)** mitochondrial area at 40000× magnification. (n=2) **(F)** Western blots of DRP1, OPA1, and MFN2. (n=6) The protein expression of **(G)** DRP1, **(H)** OPA1, and **(I)** MFN2 according to densitometry analysis. *, *p* < 0.05, **, *p* < 0.01, ***, *p* < 0.001. Data represent the mean, and error bars represent SEM. Scale bar on TEM 10000× micrographs, 2 μm; scale bar on TEM 40000× micrographs, 0.5 μm. NAFLD, non-alcoholic fatty liver disease; N, normal diet; H, high fat diet; HE, high-fat diet plus exercise; M, mitochondria; LD, lipid droplet. ns, not significant.

Mitochondrial morphology and dynamics are important to maintain normal function (23). We evaluated mitochondrial damage by examining hepatic mitochondrial morphology and integrity in high-fat diet induced NAFLD zebrafish. As shown in **Figure 3A**, liver mitochondria in the H group displayed severe fragmentation, with increased numbers of mitochondria observed (**Figure 3B**) with a smaller average diameter (**Figure 3C**), length (**Figure 3D**), and area(**Figure 3E**). Lipid droplets were clearly visible under TEM in the livers of the fish in group H. Swimming exercise significantly decreased mitochondrial numbers in the HE group compared to that in the H group. Since mitochondrial morphology is dependent on the dynamic balance between fusion and fission, the expression of fusion and fission markers was also examined (**Figure 3F**). A high-fat diet significantly disrupted mitochondrial dynamics in zebrafish, resulting in the downregulation of DRP1 (**Figure 3G**), OPA1 (**Figure 3H**), and MFN2 (**Figure 3I**), whereas swimming exercise inhibited this downregulation and alleviated the effect of a high-fat diet on mitochondrial dynamics. These data suggest that swimming exercise could maintain the dynamic balance of mitochondria in diet-induced NAFLD model zebrafish liver tissue.

### 3.4 Swimming exercise alleviates mitochondrial dysfunction in diet-induced NAFLD zebrafish

**Figure 4:**
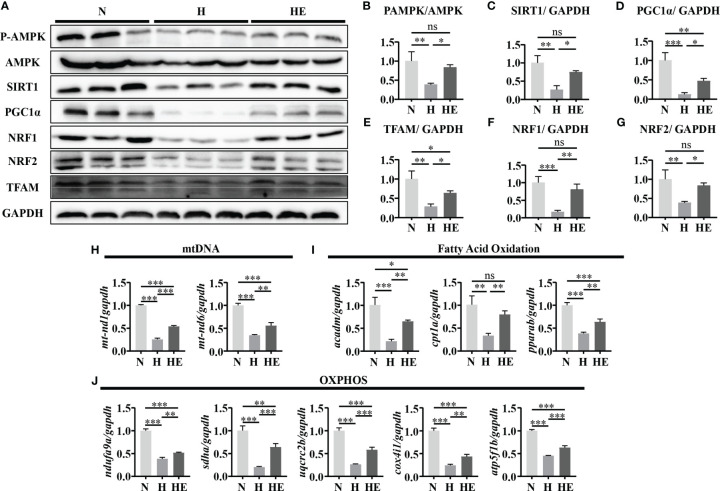
Swimming exercise alleviates mitochondrial dysfunction in diet-induced NAFLD model zebrafish livers. **(A)** Western-blot of P-AMPK, AMPK, SIRT1, PGC1α, NRF1, NRF2, and TFAM. (n=6) The protein expression levels of **(B)** P-AMPK/AMPK, **(C)** SIRT1, **(D)** PGC1α, **(E)** NRF1, **(F)** NRF2, and **(G)** TFAM according to densitometry analysis. **(H)** *Mtnd1* and *mtnd6* mRNA expression. (n=6) **(I)** The mRNA expression levels of genes related to fatty acid oxidation. (n=6) **(J)** The mRNA expression levels of genes related to mitochondrial respiratory complex subunits. (n=6) *, *p* < 0.05, **, *p* < 0.01, ***, *p* < 0.001. Data represent the mean, and error bars represent SEM. NAFLD, non-alcoholic fatty liver disease; N, normal diet; H, high fat diet; HE, high-fat diet plus exercise; OXPHOS, oxidative phosphorylation; Mitochondrial NADH dehydrogenase 1,*mtnd1;* Mitochondrial NADH dehydrogenase 6, *mtnd6.* Acyl-CoA dehydrogenase medium chain, *acadm;* Carnitine palmitoyltransferase 1A, *cpt1a;* Peroxisome proliferator-activated receptor alpha b, *pparab*); NADH:ubiquinone oxidoreductase subunit A9a, *ndufa9a*; Succinate dehydrogenase complex, subunit A, *sdha*; Ubiquinol-cytochrome c reductase core protein 2b, *uqcrc2b*; cytochrome c oxidase subunit 4I1, *cox4i1*; ATP synthase F1 subunit beta, *atp5f1b*. ns, not significant.

We next determined if swimming exercise can alleviate mitochondrial dysfunction in high-fat diet zebrafish livers. Mitochondrial biogenesis maintains the homeostasis of mitochondrial mass and function (24). We therefore quantified the expression of P-AMPK/AMPK, SIRT1, and PGC1α and downstream targets (NRF1, NRF2, and TFAM) to evaluate the effect of exercise on diet-induced NAFLD in zebrafish. The expression of these biogenesis markers was significantly downregulated in the H group compared to in the N group (**Figures 4A–G**). Similarly, *mtnd1* and *mtnd6* mRNA expression were reduced in the H group compared to in the N group (**Figure 4H**). In contrast, swimming exercise activated the AMPK/SIRT1/PGC1α pathway and thus facilitated mitochondrial biogenesis in the HE group. We assessed the expression of genes related to fatty acid oxidation and OXPHOS. Oxidative genes (*acadm*, *cpt1a*, and *pparab*) and mitochondrial respiratory complex subunits genes (*ndufa9a*, *sdha*, *uqcrc2b*, *cox4i1*, and *atp5f1b*) were significantly reduced in the H group compared to in the N group, while swimming exercise remarkably alleviated this reduction in the HE group (**Figures 4I, J**). These data suggest that swimming exercise can facilitate mitochondrial biogenesis and function in NAFLD zebrafish livers.

### 3.5 Swimming exercise restores mitophagy in diet-induced NAFLD model zebrafish

**Figure 5:**
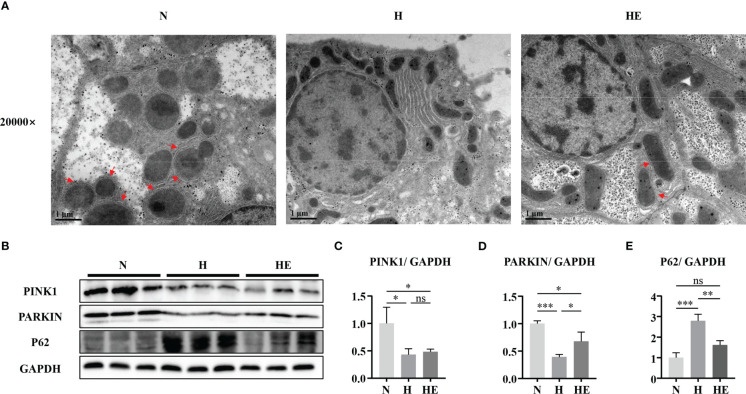
Swimming exercise restores mitophagy in diet-induced NAFLD model zebrafish livers. **(A)** Representative TEM of zebrafish liver. **(B)** Western-blot of PINK1, PARKIN, and p62 (n=6). Protein expression levels of **(C)** PINK1, **(D)** PARKIN, and **(E)** p62 according to densitometry. * *p* < 0.05, ** *p* < 0.01, *** *p* < 0.001. Data represent the mean, and error bars represent SEM. Scale bar on TEM 20000× micrograph, 1 μm. The red arrows indicated the mitophagosomes. NAFLD, non-alcoholic fatty liver disease; N, normal diet; H, high fat diet; HE, high-fat diet plus exercise. ns, not significant.

Mitophagy is vital for maintaining mitochondrial quality, and impaired mitophagy could lead to the accumulation of damaged mitochondria. As shown in **Figure 5A**, electron microscopy analysis revealed that zebrafish fed a high-fat diet lack mitophagosomes, while swimming exercise restores them. The levels of PINK1 and PARKIN were reduced, while the level of p62 was increased, in H group zebrafish livers compared to in the N group, but this effect on PARKIN and P62 was reversed in zebrafish in the HE group (**Figures 5B–E**). These results indicate that swimming exercise could activate mitophagy during NAFLD-induced liver injury.

## 4 Discussion

The present study demonstrated that swimming exercise could improve pathological changes in high-fat diet-induced NAFLD model zebrafish livers. Swimming exercise decreased the NAS score, inflammation, fibrosis content, and ROS in the livers of high-fat-diet zebrafish, and could therefore be a critical mediator of mitochondrial quality, including morphology, dynamics, and function. The protective effects of swimming exercise on mitochondrial quality in NAFLD are linked to the amelioration of liver pathology, which is supported by the observed decrease in NAS score and ROS, and increase in fatty acid oxidation and OXPHOS.

Our previous study reported that swimming exercise alleviated hepatic steatosis by inhibiting the expression of lipogenic genes and increasing the expression of fatty acid oxidation genes (16). In the present study, we confirmed the protective role of swimming exercise in a zebrafish model of NAFLD. NASH is regarded as a severer stage of NAFLD, and its progression is closely related to inflammation and oxidative stress (25, 26). The liver inflammatory response is an important driver of disease progression, which contributes to the development of NASH and liver fibrosis in NAFLD and eventually leading to cirrhosis (27). Consequences of increased ROS production during NAFLD include reprogramming of lipid metabolism in the liver, changes in insulin sensitivity, and accumulation of inflammation through interaction with innate immune signals (14). These effects suggest that oxidative stress plays an essential role in the development and progression of NAFLD. Swimming exercise suppressed inflammation (IL-1β) and fibrosis markers (COL1A1, ACTA2), and activated the expression of a key anti-inflammatory mediator, IL-10. Swimming exercise also protects the liver from inflammation and fibrosis development, and alleviates oxidative stress in diet-induced NAFLD model zebrafish livers.

Continuous mitochondrial fission and fusion occur in the liver (28). Under pathological conditions, unbalanced or disrupted mitochondrial dynamics could cause abnormal mitochondrial morphology (4). In NAFLD, the liver mitochondria often have a damaged ultrastructure with a lack of cristae and abnormal morphology, typically appearing to be smaller and fragmented (23). Previous studies have shown that impaired fusion and excessive fission are responsible for this abnormal morphology (8, 29, 30). DRP1 plays an important role in the process of mitochondrial division. In animal models of NAFLD, DRP1 protein expression is increased, indicating mitochondrial disruption (8, 31). Inhibition of mitochondrial division has a protective effect on hepatic steatosis, while alleviating HFD-induced oxidative stress and liver damage (32). During mitochondrial fusion, MFN1/2 and OPA1 integrate the outer membrane (OMM) and inner membrane (IMM) of mitochondria, respectively. Mitochondrial fusion is triggered by energy demand and stress, which can up-regulate metabolic capacity and repair damaged mitochondria (33). Mitochondrial disruption due to hepatocellular specific loss of MFN2 exacerbated NAFLD progression, inflammation, and hyperglycemia in mice fed with a high fat diet (34). These suggests that intervention of mitochondrial fusion and mitochondrial division may be an important way to improve NAFLD. In the present study, in response to a high-fat diet, NAFLD zebrafish liver mitochondria also exhibited obvious fragmentation with decreased OPA1, DRP1 and MFN2 protein expression. And swimming exercise alleviated this downregulation of the proteins with larger mitochondria. These results suggested that swimming exercise could prevent the impaired mitochondrial dynamics induced by high-fat diet in NAFLD zebrafish liver.

Mitochondrial biogenesis maintains mitochondrial mass to preserve energy homeostasis and function (35), and AMPK/SIRT1 signaling is an upstream regulator of mitochondrial biogenesis (36). Peroxisome proliferator–activated receptor gamma coactivator 1a (PPARGC1A or PGC1α) and nuclear respiratory factor 1/2 (NRF1/2) also regulate mitochondrial biogenesis (37). TFAM is a downstream target of NRF1/2, and is one of the most abundant mitochondrial DNA-binding proteins, where it controls mtDNA replication, transcription, and packaging (38). In the present study, the AMPK/SIRT1/PGC-1a axis was inhibited in NAFLD model zebrafish livers. In contrast, swimming exercise activated AMPK/SIRT1/PGC1α and ameliorated mitochondrial biogenesis by upregulating NRF1/NRF2 at the protein level, and mtDNA and mitochondrial respiratory complex subunits at the mRNA level, in NAFLD model zebrafish livers. Mitochondria are also the main site of fatty acid β-oxidation, and upregulated PGC1α could increase the expression of β-oxidation genes. NRF2 also plays a vital antioxidant role, and its activation may alleviate the oxidative stress induced by a high-fat diet (39). Swimming exercise may therefore activate mitochondrial biogenesis and improve β-oxidation and antioxidation in NAFLD model zebrafish livers.

Mitophagy is a conserved cellular process whereby dysfunctional mitochondria are selectively removed by targeting them to the autophagosome for degradation (40). Impaired mitophagy has previously been associated with NAFLD, and mitophagy-based therapy is therefore a new potential therapeutic NAFLD target (41). The PINK1-PARKIN pathway is one of the major pathways that regulates mitophagy (42). PARKIN functions as a core mitophagy-regulating protein, and can be recruited to damaged and depolarized mitochondria to induce mitochondrial clearance by mitophagy. PINK1 also plays a vital role in mitophagy by selectively accumulating on depolarized mitochondria and promoting PARKIN translocation to them (43). A recent study showed that hepatocellular specific deletion of PARKIN exacerbated fatty liver disease and insulin resistance in mice fed with a high fat diet (44). Mitochondrial autophagy defects have been reported in both high-fat diet (HFD)-induced mouse models and *in vitro* cultured cells treated with oleic acid (OA) or palmitic acid (PA), which are associated with a range of NAFLD-related phenotypes, including increased fat accumulation, elevated oxidative stress, and inflammation (45, 46). These studies suggest that removing damaged mitochondria by activating mitochondrial autophagy may be a promising approach to combat simple steatosis and NASH. In the present study, the PINK1-PARKIN pathway was inhibited, and the numbers of mitophagosomes was reduced, in NAFLD model zebrafish livers. Swimming exercise activated PARKIN protein expression and repressed P62 expression, but did not affect PINK1 protein expression. Functionally, swimming exercise alleviated mitophagosome formation in high-fat diet-induced NAFLD model zebrafish. Interestingly, a recent study reported that PARKIN could translocate to the mitochondria independent of PINK1 (47). And elevated P62 expression used to be seen as a marker of inhibited autophagy activity (48). Swimming exercise downregulated the P62 expression could be one of the main ways to activate mitophagy. The findings of the present study suggest that increased PARKIN expression and decreased P62 expression caused by exercise may partially restore mitophagy, although the underlying regulatory mechanisms remain unclear and could be investigated in future.

In conclusion, the beneficial effect of exercise on NAFLD is associated with improved mitochondrial function in a zebrafish NAFLD model. These findings highlight the importance of exercise for maintaining mitochondrial morphology and function, and enabling mitophagy. The present study confirms the possibility that exercise could be an effective strategy for targeting hepatocyte mitochondria as a treatment for NAFLD.

## Data availability statement 

The original contributions presented in the study are included in the article/**Supplementary Material**. Further inquiries can be directed to the corresponding authors.

## Ethics statement

All aspects of this research were conducted in accordance withthe Chinese guidelines for animal welfare and experimentalprotocols. Approval was obtained from the Animal ExperimentAdministration Committee of Hunan Normal University (Hunan,China; approval No.: 2018/046).

## Author contributions 

Y-YZ and X-bT designed and performed the study. Z-LC, BL, M-YS, XQ and LZ assisted with experimental guidance. Y-YZ wrote the manuscript. X-YP, Z-QZ, and C-FT revised the manuscript. All authors contributed to the article and approved the submitted version.
