# Supplementary material for: Exercise intervention improves mitochondrial quality in non-alcoholic fatty liver disease zebrafish
Source: Front Endocrinol (Lausanne). 2023 May 22;14:1162485. doi: 10.3389/fendo.2023.1162485 (PMC10239848; doi:10.3389/fendo.2023.1162485)
Supplement: Supplementary file 1 [file Table_1.docx]

**Table 1 Primer Sequence**

| Primer | Forward | Reverse |
| --- | --- | --- |
| *gapdh* | ATCATCTCTGCCCCAAGTGC | ACGGTCTTCTGTGTTGCTGT |
| *nd1* | AGCCATCTCAAGCCTAGCAG | ATTGTTTGCGCTACAGCTCG |
| *nd6* | CCGCCACAATTACAACCAGAC | TTTGCTTATTCAGCGGCCCTA |
| *pparab* | ATGTCCCACAATGCCATCCG | TCTGCTTGGCCAGGGTTTTC |
| *acadm* | TGGAGAAGGAGCTGGCTTTA | AAGACACTGCCTGGTGCTCT |
| *cpt1a* | GAACCTCACGCTAATCTACC | TGAAGGCATCTGGACTGG |
| *ndufa9a* | AGAGAATGGGAGACCAGCAAC | ATGTCTGCGTTAAGGTGCGA |
| *sdha* | TCAGTTCCACCCCACAGGTAT | TCTTTGGCATTGGGTGCGTA |
| *uqcrc2b* | TCCTGCTTCTCGGATTGGTG | GATGCTCCCTTGGTTGTCAGA |
| *cox4i1* | GGCAACTACGGCATTTCGTC | CGACCTTCGCAACTCCATGT |
| *atp5f1b* | AGGCTGGATCAGAGGTGTCT | TGAGCAAAGGTGGTAGCAGG |
